# Scientific Items

**Published:** 1877-07-20

**Authors:** 


### Scientific Items

SUNLIGHT.—Sunlight, as physicists have shown, is the source of all the forms of force with which we are familiar. The coal that heats our houses and moves our steam-engines is only the solidified sunshine of former ages, and all the life of the vegetable and an imal world now is due, directly or indirectly, to the same source. The bread that we eat is the gift of the sun, the clothes that we wear are woven out of his beams. Plants turn towards his light, and so should we if we had not forgotten our natural instincts in the artificial existence that we lead.—*Jour-Chem.*

PLANT ANÆSTHESIA.—In plants, M. Claude Bernard, to whom is due the credit of the discovery, has found that germination ceases under the influence of ether. He introduced water cresses, which germinate from day to day, into two precisely similar tubes. In one tube he placed a little ether. The plant therein on the following day was found not to have germinated, as the other had; but after being removed from the anæsthetic, the first went on and germinated in a natural manner. The plant had literally been put to sleep.

NEW FUNCTIONS OF THE LIVER.—B. F. Lautenbach (in *Medical Times*), in a series of experiments upon the lower animals, by ligating the portal vein and injecting poison into the system, has discovered that,

1*st. The liver has, for one of its functions, the office of destroying certain of the organic poisons.*

2*nd. A poison is being constantly formed in the system of every animal, which it is the office of the liver to destroy*—shown by the fact that narcotic symptoms and stupor, followed by speedy death, results from ligating the vena porta.

THE MICROSCOPE.—It is believed that we have already reached the limit of the magnifying power of the microscope, by reason of spectral images caused by diffraction of light. Whenever we exceed a magnifying power of seven or eight hundred diameters, the diffraction confuses and renders unreliable the appearance of the objects examined.

STILL BURNING.—Fifty years ago the coal in a Belgian coal mine was accidentally ignited, and though frequent efforts have been made to extinguish it, the burning still continues unabated. What vast underground caverns must have resulted from the long continued combustion of underground material.

AIR IN THE GROUND.—It has been shown by recent observations that the atmosphere permeates freely into all dry soils, and that even gas may find its way from a leaky pipe through the earth from considerable depths into dwelling houses, a fact which is important in a sanitary point of view.

VITALITY OF SNAILS.—A writer in the American Naturalist relates an instance of a snail—of the species, Helix Veatchii, which lived without food from 1859 to 1865.

ANTIHYDROPIN is a crystaline substance obtained from that hateful insect the cockroach, which, in Russia, is used as a remedy for dropsy.

METEORIC STONE.—A meteoric stone which fell recently in Saxony, contained 93.04 per cent of iron, 6.16 nickel, and 0.23 phosphorus.

